# The Dynamics of Lamin a During the Cell Cycle

**DOI:** 10.3389/fmolb.2021.705595

**Published:** 2021-08-26

**Authors:** Anat Vivante, Irit Shoval, Yuval Garini

**Affiliations:** ^1^ Physics Department, The Institute of Nanotechnology and Advanced Materials, Bar Ilan University, Ramat Gan, Israel; ^2^ Scientific Equipment Center, The Mina and Everard Goodman Faculty of Life Sciences, Bar Ilan University, Ramat Gan, Israel; ^3^ Department of Biomedical Engineering, Technion Israel Institute of Technology, Haifa, Israel

**Keywords:** lamin a, cell cycle, chromatin, imagestream, live-cells imaging, nucleus organization, continuous photobleaching

## Abstract

Lamin proteins play an essential role in maintaining the nuclear organization and integrity; and lamin A, in particular, plays a major role in the whole volume of the nuclear interior. Although the nucleus is highly organized, it is rather dynamic, it affects crucial nuclear processes and its organization must change as cells progress through the cell cycle. Although many aspects of these changes are already known, the role of lamin A during nuclear assembly and disassembly as well as its underlying mechanisms remains controversial. Here we used live cells imaging and Continuous Photobleaching (CP) method to shed light on the dynamics and mechanisms of lamin A during the cell cycle, combined with imaging flow cytometry measurements, which provides the high-throughput capabilities of flow cytometry with single-cell imaging. As a major analysis tool, we used spatial correlation algorithm for allocating the distribution of lamin A, chromatin and tubulin, as well as their mutual colocalization. Furthermore, we analyzed the distribution of lamin A along the nuclear lamina and in the nucleus interior during the cell cycle. Our results indicate that at the beginning of the cell division that include prophase, metaphase and anaphase, lamin A is distributed throughout the cytoplasm and its concentration in the chromosomal regions is reduced, whereas the spatial correlation between lamin A and tubulin is increased. It implies that lamin A also disassembled in the whole cellular volume. At the telophase and early G1, lamin A is concentrated in the whole volume of the newly formed nuclei of the daughter cells and it assembles to the lamina. We also explored the functional aspects of lamin A during the cell cycle and its binding to the chromatin versus the freely diffusion form. We found that the fraction of the bound proteins of lamin A in the S phase increased, relative to the G1 phase, which means that during replication, the concentration of lamin A on the chromatin increases. All these results shed light on the function of lamin A throughout the cell cycle.

## Introduction

In recent years, a growing number of studies have revealed the hierarchical organization of chromatin in the cell nucleus and have highlighted its importance for maintaining adequate cellular functions throughout the cell cycle. The lamin proteins, which consist of lamin A/C, lamin B1, and lamin B2, play an essential role in maintaining the nuclear organization. The nuclear lamins are intermediate filament (IF) proteins (Dechat et al., 2010) that self-assemble into higher-order structures. First, they form a coiled-coil homodimer. Next, the lamin dimers associate in a head-to-tail fashion and interact in an antiparallel fashion to form filaments (Heitlinger et al., 1992). The most prominent structure assembled from the lamins is the nuclear lamina, which is found at the inner face of the nuclear envelope membrane (Gerace et al., 1978; Krohne et al., 1978). However, lamins, and especially lamin A, are located not only at the nuclear periphery but also in the nuclear interior (Broers et al., 1999).

Among the lamin proteins, lamin A was found to play a significant role in affecting the nuclear properties, including its dynamics, elasticity, and organization (Bronshtein et al., 2015; Vivante et al., 2019; Vivante et al., 2020). Aberrations in lamins lead to numerous diseases known as laminopathies (Broers et al., 2006). Although the nucleus and lamina are highly organized, better understanding their dynamics is fundamental to understand the nuclear mechanisms and how they function (Vivante et al., 2017). Furthermore, the nuclear structure and functions change as cells progress through the cell cycle.

Many studies of the nuclear organization have been conducted; most have focused on lamin A during interphase. Prior work on the dynamics of the nucleus emphasized the crucial role of lamin A as a nuclear organizer and suggested a model of chromatin cross-linking by lamin A proteins (Bronshtein et al., 2015). However, the dynamics and mechanisms underlying lamin A during the cell cycle have been studied much less and they remain unclear.

During interphase, the lamins are located in the nuclear lamina, where they interact with inner nuclear membrane proteins and chromatin; however, a non-negligible fraction of the proteins, especially lamin A, remains in the nuclear interior and interacts with the chromatin (Bronshtein et al., 2015). At the beginning of cell division the nuclear envelope breaks down; in the early stages of mitosis, lamins are distributed throughout the cytoplasm in a diffusible (nonpolymerized) state (Moir et al., 2000a). In contrast, at the end of mitosis, the nuclear lamins assemble in daughter cells to form the nuclear lamina during nuclear envelope formation. In addition, lamins are post-translationally modified by phosphorylation. Phosphorylation leads to lamin A disassociation and depolymerization of the lamina (Peter et al., 1990; Huguet et al., 2019), which is driven by cyclin-dependent kinase (Cdk) 1 and protein kinase C (PKC) at specific sites at the beginning of mitosis (Dessev et al., 1990; Heald and McKeon, 1990). Subsequently, dephosphorylation of the mitotic sites by protein phosphatase 1a is required for lamin/lamina assembly during the telophase/early G1 transition (Thompson et al., 1997). Different phosphorylation combinations on lamin A yield markedly different effects on its assembly, subunit turnover, and mobility (Kochin et al., 2014).

Regarding nuclear assembly and disassembly, the role of the A- and B-type lamins remains controversial. The structure and organization of nuclear lamins undergoes several changes during the cell cycle (Moir et al., 2000b). Several studies claim that the interactions of lamins with chromatin and the nuclear envelope components occur very early during nuclear assembly following mitosis (Lourim and Krohne, 1993; Greenberg et al., 1996), whereas others claim that the lamins are not required for membrane assembly and are imported into the nucleus later on after the nuclear membrane is formed (Meier et al., 1991). Other studies have shown that nuclear envelope disassembly is a stepwise process in which the microtubules play an important part (Georgatos et al., 1997).

In addition, the A- and B-type lamins exhibit different assembly and disassembly pathways. Whereas lamin B1 is concentrated at the surface of the chromosomes during the anaphase-telophase transition, lamin A’s association with the nucleus begins only after the major components of the nuclear envelope, including the pore complexes, are assembled in daughter cells (Moir et al., 2000a). Another study has shown that lamin A levels in the nuclear interior gradually decrease within ∼200 min after early G1 due to relocation of the majority of lamins to the nuclear periphery (Naetar et al., 2021). The timing and patterns of disassembly also differ between these isotypes (Georgatos et al., 1997). The different disassembly process rates could be due to cell-type-specific differential expression of lamin isotypes (Rober et al., 1989).

It is common in the field to study lamin A and lamin C together, because these two alternative splice products are considered to have no significant difference between their function. However, several studies have found that the two isoforms are not equivalent in different biological aspects (Al-Saaidi and Bross, 2015). It was also found that lamin A and lamin C have different localizations as cells exit mitosis (Wong et al., 2020). Whereas lamin A is associated with the nuclear envelope during telophase, lamin C remains in the interior surrounding nucleoplasmic Lamina Associated Domain (LAD) clusters.

We use live cells imaging and multispectral imaging flow cytometry in order to shed light on the dynamics and mechanisms of lamin A throughout the cell cycle and to resolve the discrepancy mentioned above. The rarity of mitotic events within asynchronously dividing populations necessitates the use of such high-throughput methods to obtain a statistically relevant number of cells for analysis. The multispectral imaging flow cytometry method combines the high-throughput capabilities of conventional flow cytometry with single-cell imaging (Brown and Wittwer, 2000; Filby et al., 2011). Studying asynchronously dividing populations is preferable because mitotic blockers and other biomechanical drug arrests can damage the cytoskeletal organization and differentially affect the cells (Panet et al., 2015).

We measured the distribution of lamin A throughout the cell cycle, especially relative to the chromatin and the tubulin distributions. In order to determine the localization of each protein, as well as the colocalization of pairs, we used correlation methods as explained below. Furthermore, we used Continuous Photobleaching (CP) method to investigate the ratio of the free fraction to the bound fraction of lamin A protein in the G1 and S phases. Therefore, we quantified not only its distribution but also its binding properties in the nucleus interior at each phase.

## Materials and Methods

### Cell Culture

In the multispectral imaging flow cytometry measurements we used Mouse Embryonic Fibroblast (MEF) cells (the MEF cells were kindly provided by Prof. Susana Gonzalo from Saint Louis University School of Medicine, St. Louis, MO, United States) and in CP measurements we used mouse fibroblast (MF) cells with endogenous lamin A/C labeled with mEos3.2 (the MF cells were kindly provided by Prof. Roland Foisner from Max Perutz Labs, Medical University of Vienna, Vienna, Austria). The labelling procedure and confirmation was previously described (Naetar et al., 2021). The cells were maintained in Dulbecco’s high glucose modified Eagle’s medium (DMEM, Biological Industries, Israel) containing 10% fetal bovine serum (FBS, Biological Industries), 1% penicillin, and streptomycin antibiotics (Biological Industries), and 1% l-glutamine (Biological Industries), in an incubator at 37°C and with a 5% CO_2_ level. For CP measurements, we transiently transfected MF cells with pCNA-mcherry plasmid for labeling the S phase and mKO2-hCDT1 plasmid for labeling the G1 phase (the plasmids were provided by the Yaron Shav-Tal and Amit Tzur labs, Bar-Ilan University, Ramat Gan, Israel).

### Immunofluorescence for ImageStream

MEF Cells were dissociated with trypsin (Biological Industries), washed with PBS (Biological Industries), and fixed with 4% paraformaldehyde at room temperature (20 min), followed by extraction in 0.5% Triton X-100 in PBS. The cells were then blocked in 1% BSA for 30 min at room temperature. Next, the cells were incubated with anti-lamin A and anti-tubulin primary antibodies (Lamin A: Abcam, ab26300, dilution 1:300, Alpha-Tubulin: Abcam, ab7291, dilution 1:100) in blocking medium overnight at 4°C. Lamin A antibody label mainly lamin A and should not detect lamin C; The immunogen peptide used to produce ab26300 corresponds to a sequence within aa 567–664 of Human lamin A. The sequence of lamin C differs from the sequence of lamin A between aa 567–572 and aa 573–664 is missing in lamin C. The cells were washed with PBS and then incubated with secondary antibodies (Anti-rabbit Alexa Fluor-488: Abcam, ab150077, dilution 1:1,000, Anti-mouse Cy3: Abcam, ab97035, dilution 1:1,000) in blocking medium for 1 h at room temperature. Finally, DNA was stained with Hoechst 33258 (Sigma-Aldrich).

We labeled lamin A, tubulin, and chromatin. Although we refer to Hoechst as a stain for chromatin it actually labels the DNA-protein complex as Hoechst intercalates into the DNA. Tubulin was selected because it is one of the major components of the cytoskeleton. α- and β-tubulin polymerize into dynamic microtubules that function in many essential cellular processes, including mitosis (Meunier and Vernos, 2012). When cells progress from interphase to mitosis, large, relatively stable microtubule arrays are broken down and reorganized into much more dynamic mitotic spindles. The dynamics of the microtubules throughout the cell cycle have been studied in depth (Belmont et al., 1990; Walczak et al., 2008), and an interaction between the mitotic spindle and the lamin-A/C–LAP2α–BAF1 protein complex has been described (Qi et al., 2015). Therefore, we used tubulin and chromatin labelling to determine the cell cycle stage and the localization of tubulin and chromatin relative to lamin A.

### Multispectral Imaging Flow-Cytometry Analysis

Cells were imaged using multispectral imaging flow cytometry (ImageStreamX mark II imaging flow-cytometer; Amnis Corp, Seattle, WA, United States). For asynchronously dividing cells, the percentage of the mitotic cells is around 1.5–2. Therefore, a minimum of 4  ×  10^4^ cells were collected from each sample and hundreds of cells were identified for each cell cycle stage. The data were analyzed using an image analysis software (IDEAS 6.2; Amnis Corp.) and the MATLAB programs that we developed. Images were compensated for fluorescent dye overlap by using single-stain controls. The serial gating strategy used to identify the mitotic cell population was as follows: Cells were first gated for single cells using the area and aspect ratio features on the brightfield (BF) image (the aspect ratio is the ratio of the minor axis to the major axis; it describes how round or oblong an object is). Uncropped cells were gated using the centroid X (the number of pixels in the horizontal axis from the upper left corner of the image to the center of the mask) and the area features. Focused cells were gated using the Gradient RMS feature (Blasi et al., 2016). Next, the BF area vs the nuclear intensity (Hoechst) was plotted and the extreme levels of nuclear intensities were gated out. In order to gate the mitotic cells, the mean vs the max pixels of the lamin A marker was plotted. As lamin A spreads throughout the dividing cell, in contrast with a peri-nuclear distribution in non-dividing cells, the mitotic cells were recognized as having a low max pixel intensity of lamin A. The mean and max pixel intensities were calculated over a combined mask that covers the peri-nuclear area and was created as followed: A morphology mask of the Hoechst stain eroded by 2 pixels was subtracted from a morphology mask of the Hoechst stain dilated by 1 pixel.

To further subdivide them into the specific cell division phases, we gated them according to nuclear morphology based on the spot count and aspect ratio intensity features in the lamin A image. We identified prophase cells (a high nuclear aspect ratio with a single nuclear spot) and metaphase cells (a low nuclear aspect ratio with a single nuclear spot) and anaphase cells (a low nuclear aspect ratio with two nuclear spots). The mask for the nuclear spot count was created as follows: a threshold mask of the 60% of the intensity rate combined with a range mask to exclude signal noise. The telophase population was derived from the non-mitotic cells and identified as having a low nuclear aspect ratio with two nuclear spots.

### Correlation Analysis

We adopted a correlation analysis method that is best suited for describing the co-localization of the pairs of features. We calculated the Pearson correlation between each pair of images; it computes the correlation coefficient between two images using the equation:

$$r=\;\frac{\sum_{m}\sum_{n}\left(L_{\mathrm{mn}}-\bar{L}\right)\left(C_{\mathrm{mn}}-\bar{C}\right)}{\sqrt{(\sum_{m}\sum_{n}{\left(L_{\mathrm{mn}}-\bar{L}\right)}^{2}\left(\sum_{m}\sum_{n}{\left(C_{\mathrm{mn}}-\bar{C}\right)}^{2}\right)}}$$

where $L_{\mathrm{mn}}$ is the intensity of lamin A in pixel mn, $C_{\mathrm{mn}}$ is the intensity of chromatin or tubulin in pixel mn, $\bar{L}$ is the mean intensity of lamin A, and $\bar{C}$ is the mean intensity of chromatin or tubulin. The pixels that were taken into account are only those that are in the cell area (and not in the background). The mask of the cell area was determined using a threshold in the lamin and tubulin channels. The correlation coefficient values can range from –1 to +1, where a value of around +1 indicates a positive and strong correlation, whereas values approaching –1 indicate a strong negative correlation. A value around 0 indicates that there is no specific correlation. We followed this correlation for each of the measured cells.

We also calculated the Spearman correlation between each pair of images and obtained similar results. The Spearman correlation assesses monotonic relationships (whether linear or not), whereas Pearson’s test assesses the linear correlation. This indicates that the correlation that we found is not only monotonic, but also linear.

### Continuous Photobleaching

CP measurements were performed as described previously (Bronshtein et al., 2015; Vivante et al., 2017). Briefly, CP is especially useful for determining the ratio of the free-to-bound fraction of a protein in a cell (Wachsmuth et al., 2003); some of the proteins freely diffuse, whereas others are mostly bound. Often, the two fractions exist (free and bound proteins) at each point in the cell or only in specific regions. For CP measurements, a confocal microscope set-up is used; after the cell image is measured, the laser is “parked” in a selected point in the image, and the fluorescence intensity is measured for ∼60 s. The measured spot size is diffraction-limited with a typical diameter of ∼180 nm. During the measurement, the excitation laser slowly bleaches the fluorescent proteins (FP); therefore, the fluorescent signal is proportional to the number of unbleached FPs that remain in the optical volume. The free fraction of the molecules diffuses through the measured point and their intensity is only slowly bleached, leading to a slowly decreasing linear curve of the intensity as a function of time. In contrast, the bound fraction remains in the measured spot and bleaches much faster, leading to an exponential decaying intensity curve as a function of time. When the two sub-populations coexist, a freely diffusing fraction and a bound fraction, the CP curve, which shows the intensity as a function of time, starts as an exponential decay of the bound fraction and continues with a slowly decreasing linear curve of the freely diffusing fraction. These can be resolved as explained below.

The CP was analyzed using a MATLAB program that we developed. We normalized the intensity (so that the peak intensity is equal to 1) and the measured data was first smoothed within a 0.1 s window. Then, we fit the curve to a linear combination of an exponentially decaying function and a linear curve. The ratio of the value of these two functions at *t = 0* gives the ratio of the free/bound fractions. The experiment starting point, when the laser was turned on (*t = 0*), was determined based on the maximal derivate of the intensity trace. The intensity trace was fitted to the model equation $I\left(t\right)=ae^{-\mathrm{bt}}+\mathrm{ct}+d$ and the ratio of (free proteins)/(total number of proteins) was calculated as the ratio between the extrapolated value of the intensity linear term, the intensity of the free fraction at t = 0 which is equal to d, and the intensity value at the beginning of the measurement, I(0)=a+d. This may be more complex, however, if the bound particles have a binding-unbinding time, which is in the time range of the measurement. In such a case, a more accurate dynamic set of equations has to be solved (Wachsmuth et al., 2003; Bronshtein et al., 2015). The CP method provides a rather simple and accurate information on the protein dynamics, and the ratio of the bound-to-free fractions of the protein.

For imaging, we used a Leica confocal microscope, TCS SP8 SMD. Imaging was performed with a HC PL APO 63x/1.20 W CORR objective lens for 60 s. Cells were placed in a 37 C incubator (LCI, Seoul, Korea) while maintaining a 5% CO_2_ level. For the confocal images, a HeNe (633 nm) laser was used. For bleaching, a PicoQuant Laser (640 nm) was used with an optimal optical power of ∼0.7 µW. The signal was detected by a highly sensitive HyD SMD detector.

## Results

**FIGURE 1:**
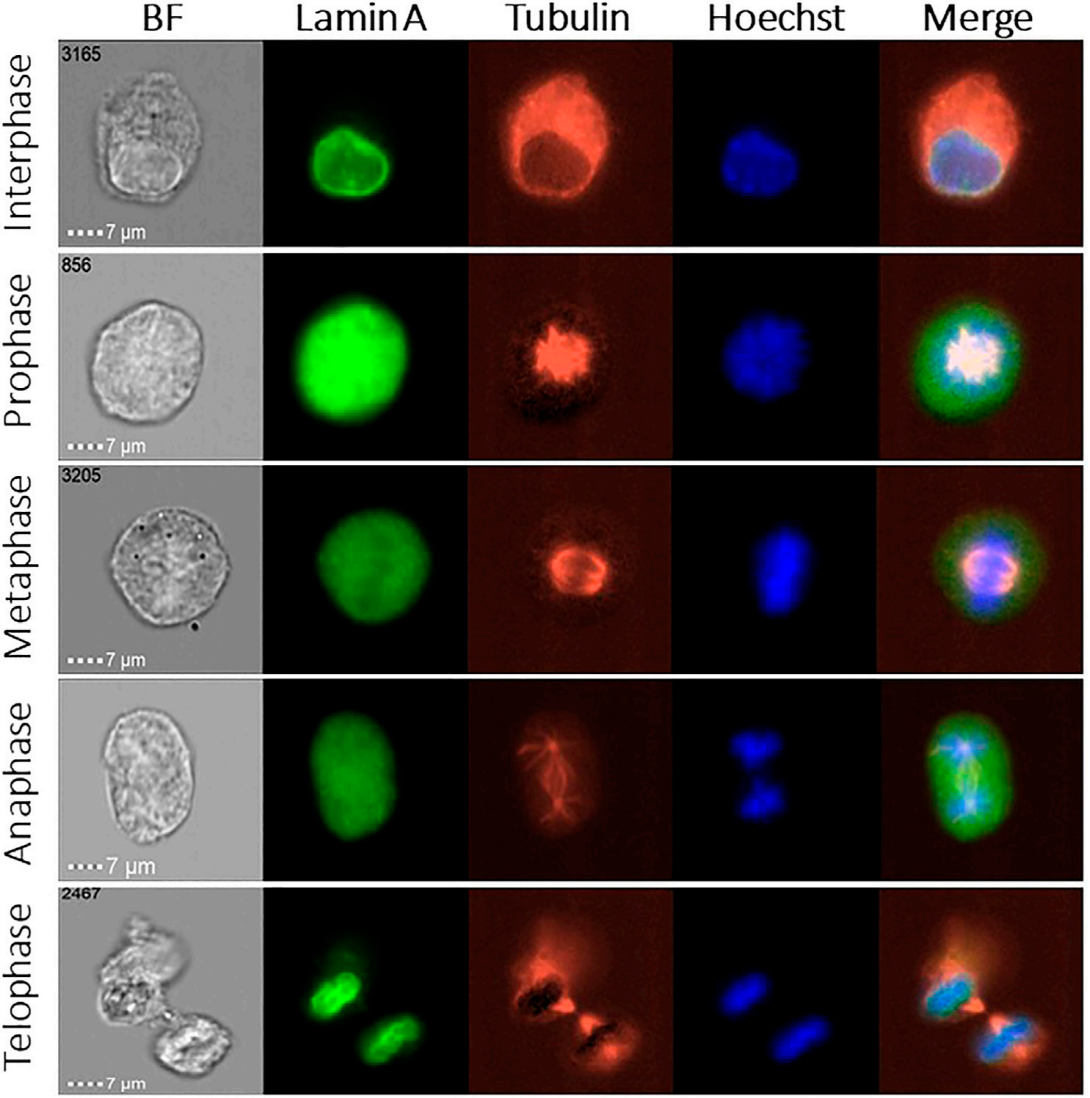
ImageStream images of the cell cycle phases in MEF cells. Representative fluorescence images of lamin A (green), tubulin (red), and chromatin (blue) in 5 different stages of the cell cycle, together with the brightfield (BF) image. Merged images show the lamin A, chromatin, and tubulin localization.

We measured thousands of MEF cells in order to follow the localization of lamin A during mitosis, and its colocalization relative to the chromatin and tubulin. Representative images of each cell cycle stage are shown in Figure 1. We present the brightfield image of the cell and three different fluorescent channels: lamin A in green, tubulin in red, and chromatin in blue, as well as a merged image of three of them, for the interphase and the four mitotic phases (prophase, metaphase, anaphase, and telophase).

**FIGURE 2:**
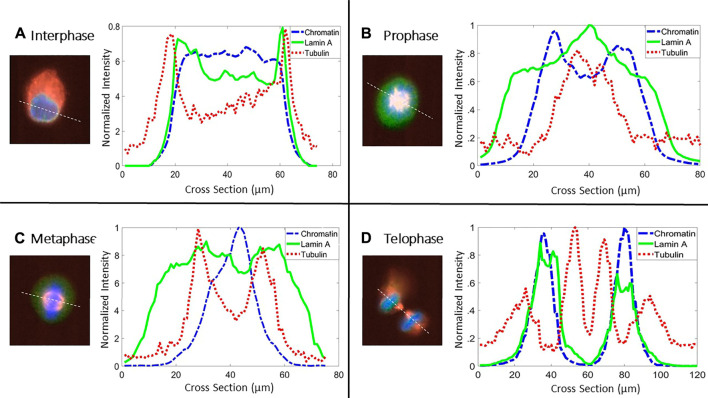
Cross section of fluorescent intensities. Representative cross-section of the intensities of lamin A (green solid line), chromatin (blue dashed line), and tubulin (red dotted line) in four different stages throughout the cell cycle in MEF cells. White dotted lines in the merged images denote the cross-section used for plotting the intensities. Line colors in the graphs are similar to the fluorescent channels in the merged image (lamin A (green), tubulin (red), and chromatin (blue)).

**FIGURE 3:**
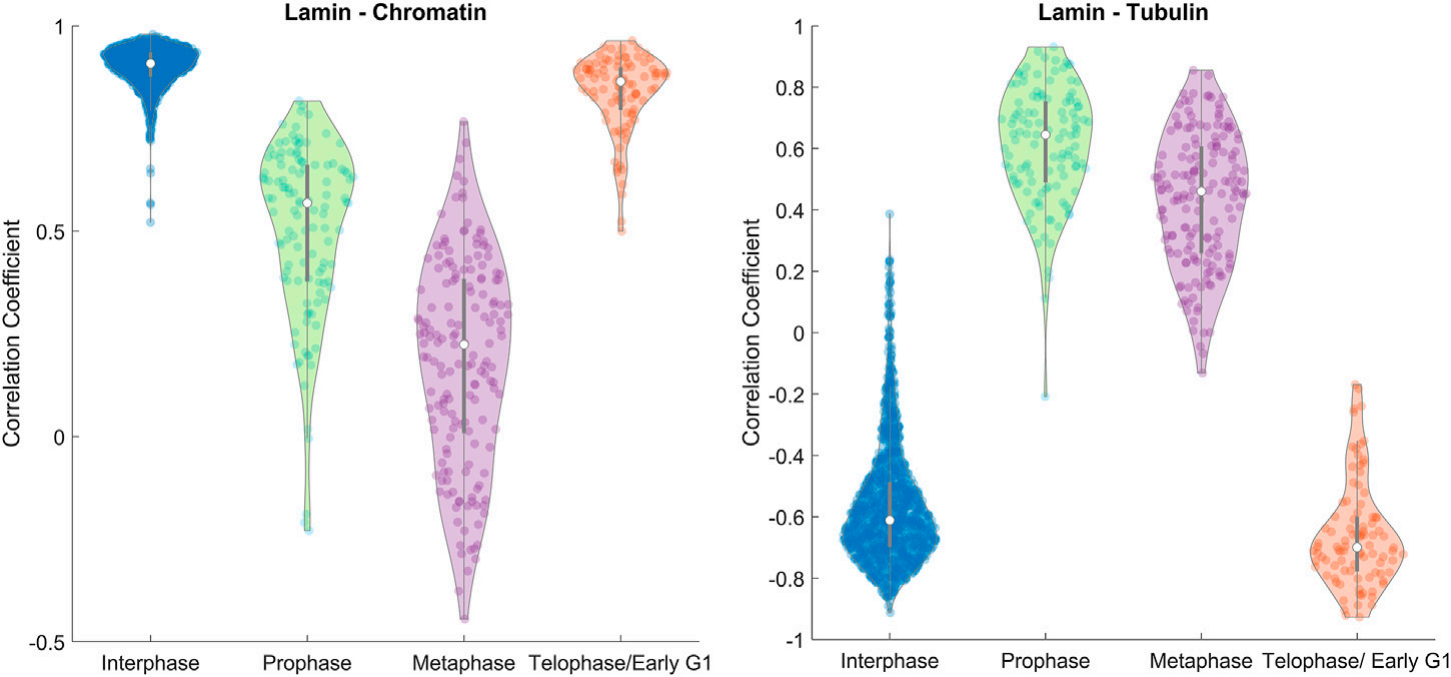
Image correlation. The distributions of the spatial correlation coefficient between lamin A–chromatin **(Left)** and lamin A–tubulin **(Right)** shown in violin plots (Bechtold, 2016) calculated for all the measured MEF cells. The violin plot shows the distribution of the correlation coefficient for different phases, the median (white circle), and interquartile ranges (thick gray bar). Data were derived from ∼1200 MEF cells in interphase, ∼100–170 MEF cells in each phase of mitosis.

**FIGURE 4:**
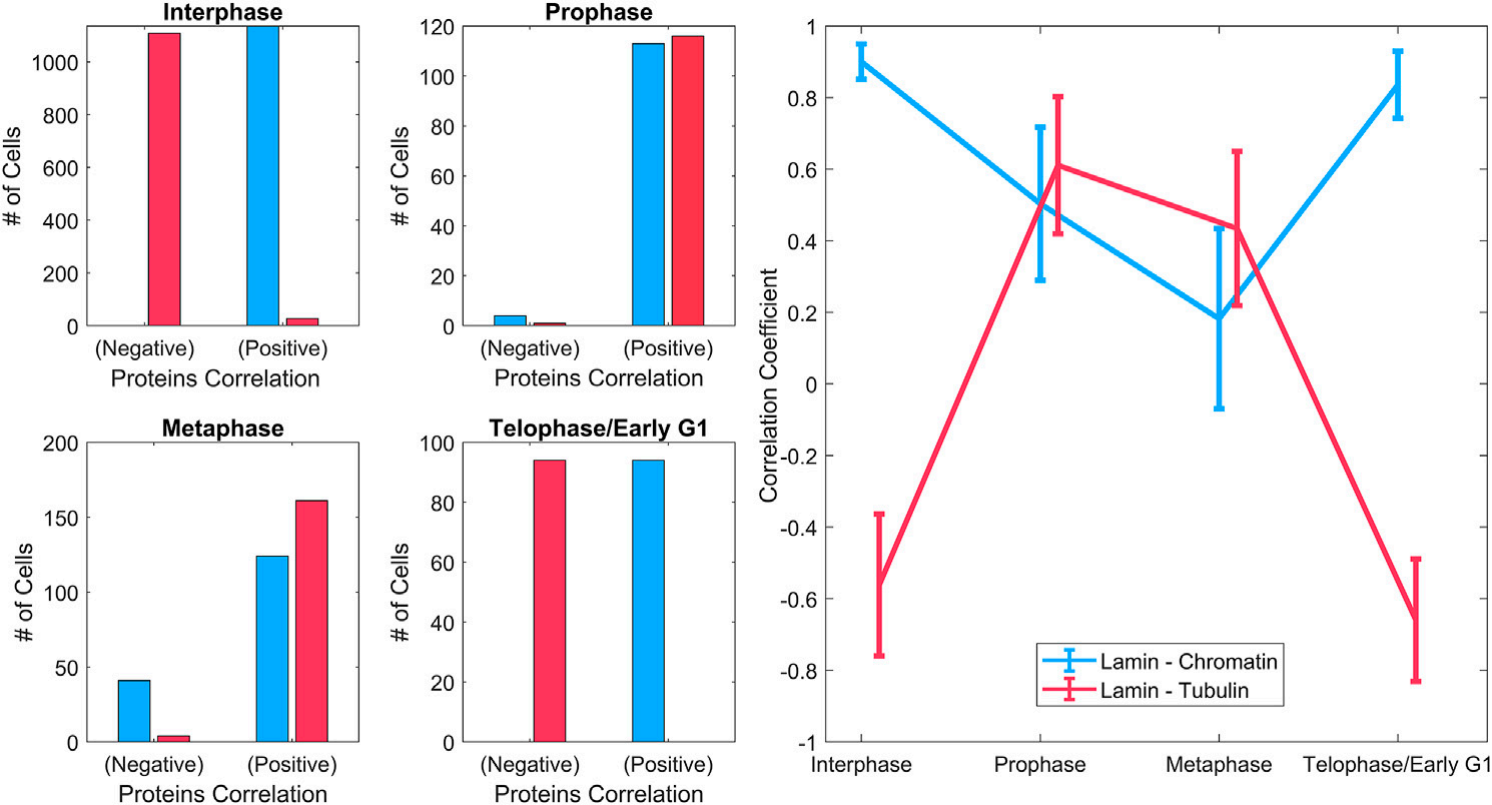
Spatial correlations of lamin A with chromatin and tubulin. **Left:** Histograms of the overall positive and negative correlation coefficient of lamin A–chromatin (blue), and lamin A–tubulin (red) shown for different phases throughout the cell cycle. **Right:** The mean correlation coefficient of lamin A–chromatin (blue) and lamin A–tubulin (red) throughout the cell cycle. It emphasizes the trendline of the correlation during the cell cycle. Data were derived from ∼1200 MEF cells in interphase and ∼100–170 MEF cells in each of the mitotic phases.

**FIGURE 5:**
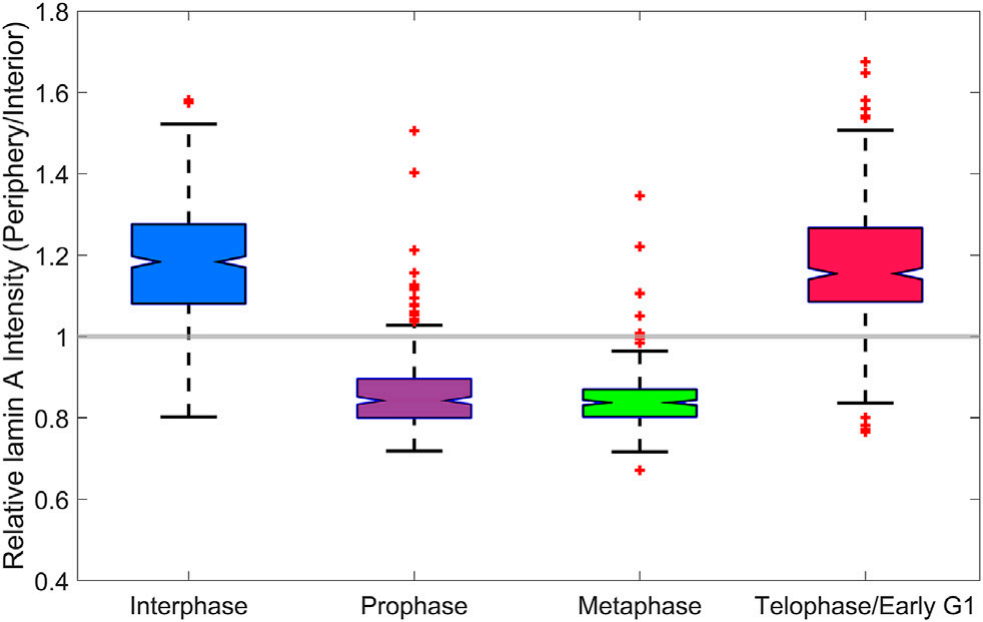
Analysis of lamin A in the lamina. A boxplot of the intensity profile of lamin A at the periphery of the nucleus, relative to the interior of the nucleus at different cell cycle stages in MEF cells. In the interphase and telophase/early G1, the relative intensity is larger than 1, whereas in prophase and metaphase, it is smaller than 1.

As one can see in Figure 1, in the early stages of mitosis, namely, prophase, metaphase, and anaphase, lamin A is distributed throughout the cytoplasm. However, lamin A’s distribution is not necessarily uniform because it is gradually reduced throughout mitosis in the region containing the chromosomes and it is increased in the tubulin region. In Figures 2–5 we present the analysis of the data, however for the anaphase we do not have enough data, therefore we decided not to include this phase.

In order to allocate the distribution of lamin A, chromatin, and tubulin throughout the cell cycle, we draw the intensities of each one of them along a diameter of the cell (Figure 2). Figure 2 shows a representative cross-section plot of the intensities of lamin A (green solid line), chromatin (blue dashed line), and tubulin (red dotted line) in different stages of the cell cycle. At interphase and telophase, lamin A and chromatin are located in the nucleus; however, lamin A is more concentrated on the lamina, which can be shown by the peaks of lamin A intensity at the borders of the chromatin. During prophase and metaphase, lamin A appears more in the tubulin region and less in the chromatin region.

To clearly demonstrate the distribution of lamin A throughout the cell cycle, relative to the chromatin and tubulin distributions, statistically on all the measured cells, we adopted a correlation analysis method that is best suited for describing the co-localization of the pairs of features (as described before in Materials and Methods section).

A violin plot of the correlation coefficient distribution for each phase is shown in Figure 3. The plots show the distribution of the data, the median (white circle), and the interquartile ranges for the lamin-chromatin correlation (Figure 3, left) and the lamin-tubulin correlation (Figure 3, right) (Bechtold, 2016). We also calculated the overall positive and the negative percentages of the correlation and summarized them for a simple characterization of the correlation (Figure 4, left), and calculated the mean correlation coefficient in order to follow the trendline of the correlation during the cell cycle (Figure 4, right).

The data show that lamin A and chromatin are both located in the nucleus during interphase, since the correlation between them is positive and approaches +1 (Figure 4, left); however, tubulin is located at the cytoplasm during interphase; therefore, the correlation between lamin A and tubulin is negative. During mitosis, lamin A is distributed throughout the cytoplasm and is gradually reduced in the region containing the chromatin; therefore, the correlation between lamin A and chromatin is reduced in prophase and continues to be reduced in metaphase. However, tubulin enters the nucleus in mitosis; therefore, the correlation between lamin A and tubulin becomes positive. In telophase, lamin A and the chromatin enter the nucleus and tubulin emerges to the cytoplasm; therefore, the correlation returns to be positive/negative, respectively.

Next, we tracked the reassembly of the nuclear lamina by lamin A, and the cell cycle stage in which reassembly process occurs. In order to demonstrate this, we analyzed the intensity profile of lamin A at the periphery of the nucleus, relative to the interior of the nucleus at each cell cycle stage. We manually distinguished between the periphery (and the nuclear lamina if appears) and the interior of the nucleus, due to a large variance of the cells size and morphology. The relative intensity (periphery divided by interior) at each cell cycle stage is shown in a boxplot (Figure 5). We added a constant gray line, which signifies a relative intensity that is equal to 1, for easily identification of the reassembly of the nuclear lamina.

As we showed before, during telophase, lamin A is concentrated together with chromatin in the newly formed nuclei of the daughter cells. Furthermore, these results indicate that during telophase/early G1, lamin A reassembles to the lamina. In interphase and telophase/early G1, the relative intensity is larger than 1, which indicates that lamin A is located mainly in the lamina and to a lesser extent in the interior of the nucleus. In contrast, in prophase and metaphase, the relative intensity is smaller than 1, which indicates that lamina is not reassembled during these stages and that lamin A is uniformly distributed in the volume of dividing cells, outside the volume of the chromatin.

**FIGURE 6:**
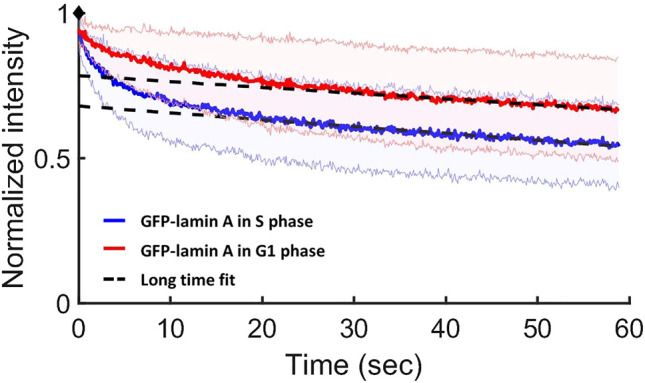
CP results for lamin A in the G1 and S phases in MF cells. Normalized CP curves of GFP-lamin A in the G1 phase (red) and in the S phase (blue). The intersection of the linear long-time fit (the dashed black line) and the *y*-axis shows the free fraction (85 ± 5% of lamin A in the G1 phase and 75 ± 5% lamin A in the S phase). The red and blue shaded areas above and below each curve refer to the STD of the measurements. Data were derived from ∼65 measurements at each phase.

In order to explore the dynamics of lamin proteins, and not only their distribution and localization, we implemented the CP method, which provides crucial information on the mobility and binding properties of the proteins. It was previously shown that during mitosis (prophase, metaphase, and anaphase) lamin A is distributed throughout the cytoplasm in a freely diffusive manner (Moir et al., 2000a). However, during interphase (the G1, S, and G2 phases), some of the proteins freely diffuse, whereas others are bound (Bronshtein et al., 2015). In both cases, freely diffuse or bound proteins, lamin A could be in monomers or dimers or short oligomers state. If there would have been lamin A filaments in the nucleus, even short ones, they should have been observed but we have never observed such filaments throughout many experiments. Using the CP method on MF cells, we explored the ratio of the free fraction to the bound fraction of lamin A protein in the G1 and S phases thereby quantifying the localization level of the protein at each phase (Figure 6).

We found that in the S phase the free fraction of lamin A is 75 ± 5%, whereas in the G1 phase the free fraction is 85 ± 5%. Therefore, our results indicate that the fraction of the bound proteins of lamin A in the S phase increased, relative to the G1 phase.

## Discussion

The localization and dynamics of lamin A during interphase have been previously studied (Bronshtein et al., 2015; Vivante et al., 2020). It was shown that lamin A is required for the 3D organization of chromosomes and for maintaining the chromosome territories in the cell nucleus. However, during mitosis and the nuclear lamina assembly process at the end of mitosis, the localization and function of lamin A remain to be elucidated.

For measuring 3D organization during mitosis, we used multispectral imaging flow cytometry. The method has the advantage of measuring thousands of cells in a 2–3 h experiment. Even though there are only ∼2% of dividing cells in the whole population, the method provided a sufficient amount of cells for having significant statistics for the study. Getting to a similar number of cells with an imaging system, will take a much longer time, probably few days. It is important not to use synchronization that would makes it easier to find mitotic cells, but it was also shown to affect the biological state of the cells (Panet et al., 2015). Nevertheless, the method also has disadvantages, the major one arises from the fact that in order to measure in flow, the cells must be maintained in suspension which is not their natural adherent form. It was shown before that cells that are being disassociated from the slide go throw biomechanical changes and that lamin A is phosphorylated (Buxboim et al., 2014). However, to overcome that in our study, the fixation was performed a short time after disassociation (less than 10 min). Furthermore, by observing the cells from our results, we note that cells that are in interphase have a well distinct lamina which assures that a significant percentage of lamin A proteins is not phosphorylated, otherwise the lamina should be completely disassembled. It can be important to repeat similar measurements with a high-throughput system that will allow to measure adherent cells. We found that during the early stages of mitosis, namely, prophase, metaphase, and anaphase, lamin A is distributed throughout the cytoplasm, but not necessarily uniformly. Lamin A is gradually reduced in the region containing the chromosomes and its concentration increases in the tubulin region. The distribution of lamin A relative to the chromatin, indicates that not only lamin A disassembles from the chromatin during mitosis, maybe due to the phosphorylation of lamin A, it also avoids the region of the chromatin for proper mitosis. The distribution of lamin A relative to tubulin, indicates their mutual affect during mitosis. The interaction between lamin A/C and the mitotic spindle has been previously described (Qi et al., 2015).

We also show that during telophase/early G1, lamin A reassembles to the nuclear lamina, which is in agreement with a previous study (Wong et al., 2020). However, other studies have shown that lamin A only gradually becomes incorporated into the peripheral lamina during the first few hours of early G1 of the cell cycle (Moir et al., 2000a; Naetar et al., 2021). These differences could be explained by variations between different cell types.

Our previous studies suggested that lamin A proteins form chromatin cross-links during interphase and therefore maintain the chromatin organization. However, the mechanism and the interactions between lamin A and chromatin during the cell cycle raise interesting open questions. During mitosis (prophase, metaphase, and anaphase), lamin A is distributed throughout the cytoplasm in a freely diffusive manner, and it is reassociated with chromatin during early G1 stage (Moir et al., 2000a). During interphase (the G1, S, and G2 phases), some of the proteins freely diffuse, whereas others are bound (Bronshtein et al., 2015).

According to our CP results, in the S phase the fraction of bound lamin A proteins is slightly increased, relative to the G1 phase. The fraction of the bound lamin A that we found in both phases is smaller than previously reported (Bronshtein et al., 2015; Vivante et al., 2019), but this is probably a result of the somewhat different experimental conditions, most likely a lower laser intensity that was used during the measurement; however, the comparison of the two phases is still valid. The higher fraction of bound proteins during the S phase is most likely due to the larger amount of chromatin in the nucleus that requires more bound lamin A for maintaining its order. This is in agreement with another study, which explored the lamina-associated domains (LADs) during the cell cycle, and showed that S phase chromatin is characterized by transiently increased lamina interactions (van Schaik et al., 2020). Previously, a study of the dynamic properties of chromatin in cells that do not express lamin A, did not find significant differences between the chromatin dynamics in G1, S, and G2 phases (Bronshtein et al., 2016). However, depletion of lamin A results in a significant increase in the chromatin dynamics in all cell cycle stages; therefore, this small difference in the bound fraction of the proteins between the G1 and S phase is neglected here.

Taken together, Lamin A proteins form chromatin cross-links during interphase in the whole nuclear volume, which are widespread throughout the genome. Together with the chromatin binding to the lamina, these mechanisms are crucial for chromatin organization. The number (and density) of the cross-links in the nuclear interior is increased during DNA replication, as it is necessary to maintain the order of a double amount of chromatin in this phase. At the beginning of mitosis, lamin A phosphorylation leads to disassembly of the lamina from the chromatin. Our findings that during mitosis there is less lamin A in the chromatin regions suggests that phosphorylation also leads to the disassembly of lamin A in the whole cellular volume. Finally, during telophase/early G1 lamin A is reconnected to the chromatin, the lamina reassembles, and chromatin regains its stable form that maintains its organization. This finding, which correlates with few of the previous publications (Bronshtein et al., 2015; Bronshtein et al., 2016; Vivante et al., 2019; Vivante et al., 2020), emphasizes the importance of lamin A in the organization of the genome as soon as the two new daughter cells are formed. Lamin A may have an effect on the cellular function that is not related directly to the chromatin organization; this is an interesting question that should be studied in the future. For future work, it will be interesting to continue study the distribution of other nuclear related proteins, such as lamin B, BAF and emerin.

In this study we combined single cell imaging with the high-throughput capabilities of conventional flow cytometry; this allows us to use asynchronously dividing cells for more natural and reliable results. We explored the localization and interaction between lamin A and chromatin during the cell cycle phases, in order to shed light on the mechanism and dynamics of lamin A during the cell cycle and to reveal more details about this controversial issue. Our findings emphasize the significance of lamin A and chromatin interactions in the nucleus interior throughout the cell cycle (interphase and mitosis) for maintaining cell organization and function.

## Data Availability

The raw data supporting the conclusions of this article will be made available by the authors, without undue reservation.
